# Rab23 is a flagellar protein in *Trypanosoma brucei*

**DOI:** 10.1186/1756-0500-4-190

**Published:** 2011-06-15

**Authors:** Jennifer H Lumb, Mark C Field

**Affiliations:** 1 Cambridge Institute for Medical Research, MRC/Wellcome Trust building, Addenbrooke's Hospital, Hills Road, Cambridge, CB2 0XY, UK

**Keywords:** Rab23, flagellum, trafficking, trypanosome, IFT

## Abstract

**Background:**

Rab small GTPases are important mediators of membrane transport, and orthologues frequently retain similar locations and functions, even between highly divergent taxa. In metazoan organisms Rab23 is an important negative regulator of Sonic hedgehog signaling and is crucial for correct development and differentiation of cellular lineages by virtue of an involvement in ciliary recycling. Previously, we reported that Trypanosoma brucei Rab23 localized to the nuclear envelope [1], which is clearly inconsistent with the mammalian location and function. As T. brucei is unicellular the potential that Rab23 has no role in cell signaling was possible. Here we sought to further investigate the role(s) of Rab23 in T. brucei to determine if Rab23 was an example of a Rab protein with divergent function in distinct taxa.

**Methods/major findings:**

The taxonomic distribution of Rab23 was examined and compared with the presence of flagella/cilia in representative taxa. Despite evidence for considerable secondary loss, we found a clear correlation between a conventional flagellar structure and the presence of a Rab23 orthologue in the genome. By epitope-tagging, Rab23 was localized and found to be present at the flagellum throughout the cell cycle. However, RNAi knockdown did not result in a flagellar defect, suggesting that Rab23 is not required for construction or maintenance of the flagellum.

**Conclusions:**

The location of Rab23 at the flagellum is conserved between mammals and trypanosomes and the Rab23 gene is restricted to flagellated organisms. These data may suggest the presence of a Rab23-mediated signaling mechanism in trypanosomes.

## Introduction

Rab proteins are important control elements of vesicle transport and related functions [2]. In African trypanosomes there are sixteen Rab-like proteins with various roles in exocytosis, endocytosis and possibly differentiation/life cycle progression [3-5]. Essentially members of the family have similar locations and functions to their mammalian or fungal orthologues where such orthologous relationships exist [4,6].

In contrast, amongst the Trypanosoma brucei Rab repertoire is Rab23 (TbRab23), which was localized to the inner nuclear envelope using polyclonal antibodies [1]. Significantly, mammalian Rab23 was first recognized in a murine model through genetic interactions with Sonic hedgehog (Shh), a protein crucial to development and differentiation of cell lineages [7,8]. Mammalian Rab23 localizes to the plasma membrane, endosomes, and cytosol and functions as a negative regulator of Shh [9-11].

The Shh pathway is metazoan-specific and essential for growth and organ patterning [12,13]. Several components of the pathway localize to primary cilia and intraflagellar transport (IFT) is required for Shh signaling [14-18]. Recent data indicate that Rab23 is essential for ciliogenesis, localizes to cilia in MDCK cells and functions to recycle the Shh pathway receptor, Smoothened (Smo), from the ciliary compartment [19,20].

As Shh signaling is restricted to metazoa, this raises intriguing questions about the possible function of TbRab23 in T. brucei and other non-metazoan organisms and may point towards a divergent function. Given that no Rab protein has been associated with the nucleus in any organism since our earlier publication, and accumulation of evidence for a role of Rab23 at the mammalian flagellum, we sought to re-examine the location of Rab23 in trypanosomes and its phylogenetic relationship with the presence of a flagellum. Using epitope-tagged chimeras of TbRab23 to avoid issues with antisera cross-reactivity, we now find that TbRab23 is associated with the flagellum, and not the nucleus as previously reported [1]. Further, Rab23 is only found in flagellated taxa. Importantly our data are consistent with the studies in mammalian cells and indicates that the function of Rab23 is likely conserved across the eukaryotes.

## 2. Materials and methods

### 2.1. Bioinformatics

Genomes were chosen for sampling based on three main criteria: i) proposed position in the evolutionary tree of eukaryotes [21] ensuring adequate representation of major supergroups, ii) completeness of the sequenced genome, and iii) the availability of a carefully annotated protein database. BLAST searches were conducted initially using TbRab23 (Tb10.6k15.1990), TbRab28 (Tb927.6.3040), TbRab7 (Tb09.211.2330), HsRab23 (NP_057361.3), and HsRab28 (CAA64364.1) as queries [22]. As GTPases are very highly conserved, e values of less than e^-20 ^were used as an initial cutoff. The top two hits were then reciprocally used as queries for a BLAST search against T. brucei and Homo sapiens sequence databases. Only those that retrieved the query sequence were assigned as candidate orthologues. If no clear orthologue was identified, additional searches were performed using protein sequences from closely related species. In cases where the retrieved sequence was a named orthologue of the query, reciprocal BLAST searches were not performed. The Rab5 dataset was kindly provided by Dr. Joel Dacks, University of Calgary.

Initial protein sequence alignments were produced in Clustal × [23] on the ODIN server at the University of Calgary, and manually edited in MacClade [24]. Phylogenetic reconstructions were performed by analyzing the alignment using ProtTest (V1.3) [25] to select the appropriate model of sequence evolution, and then MrBayes (V3.2) run locally for 10^6 ^generations and PhyML at http://www.atgc-montpellier.fr/phyml (100 pseudoreplicates) under WAG+I+G substitution model [26].

### 2.2. Cell lines and cell culture

Trypanosoma brucei brucei bloodstream form (BSF) and procyclic form (PCF) Lister 427 strains were maintained in HMI-9 and SDM79 medium respectively supplemented with 10% foetal calf serum (FCS), L-glutamine and penicillin/streptomycin as described previously [27]. Single Marker BSF (SMB) cells [28] were cultured in HMI-9 with the addition of geneticin to a final concentration of 2.5 μg/ml. Tetracycline-responsive PTT PCF [29] were cultured with the addition of hygromycin and geneticin, each to a final concentration of 25 μg/ml. Cells in exponential growth phase ( < 1 × 10^6^/ml and 3-8 × 10^6 ^cells/ml for BSF and PCF cells respectively) were used in all experiments.

### 2.3. Generation of transgenic parasites

Primers were designed and verified for specificity using RNAit [30] to amplify a fragment of the TbRab23 ORF from BSF and PCF genomic DNA (BSF: For- CGCTCTCGAGGAGGTGGGTTAAACGGAGTG, rev- GCTAGGATCCCGCTCGGTCCACATTC; PCF: For- GCATGGATCCAAGAGCGTTTGCCTCCGTCA, rev- GCTTAAGCTTCCGCTCCACCAGATTTCGAT). The fragments were cloned into the tetracycline-inducible p2T7^TABlue ^or the p2T7^177 ^plasmid for RNAi in BSF and PCF respectively. An AMAXA Nucleofector^® ^II was used following the manufacturer's protocol as described previously [31] to transfect tetracycline-responsive SMB cells with NotI-digested plasmid. Single clones were selected and maintained in the presence of 5 μg/ml hygromycin and 2.5 μg/ml geneticin. PTT PCF stable cell lines were generated in cytomix (pH 7.6 2 mM EGTA, 120 mM KCl, 0.15 mM CaCl_2_, pH 7.6 10 mM K_2_HPO_4_/KH_2_PO_4_, pH 7.6 25 mM HEPES, 5 mM MgCl_2_.6H_2_O, 0.5% Dextrose, 100 μg/ml BSA, 1 mM Hypoxanthine) containing 20 μg linearized DNA using a Bio-Rad Gene Pulser II set to 1.5 kv and a capacitance of 25 μF. Single clones were selected by serial dilution in medium containing 20% FCS, 25 μg/ml hygromycin, 25 μg/ml geneticin, and 3 μg/ml phleomycin.

For ectopic expression, TbRab23 was amplified from genomic DNA (For: CGGAAGCTTCGTTGAAGAGAGGTGGGTTAAAC, rev: GCTACGGGATCCCTACATAACACTGCACTTTTTCTTC). The PCR product was cloned into either pHD1034 or pXS219 containing an N-terminal HA- or YFP-epitope, for expression in BSF and PCF cells respectively. WT SMB cells or SMB cells containing the pT7^TABlue^-TbRab23 plasmid were transfected with 5 μg of linearized pHD1034-TbRab23. SMB pHD1034-TbRab23 single clones were selected and maintained in the presence of 2.5 μg/ml geneticin and 0.2 μg/ml puromycin, SMB cells transgenic for both TbRab23 RNAi and expression were selected and maintained as above with the addition of 5 μg/ml hygromycin. Twenty micrograms of linearized pXS219-TbRab23 was used to transfect WT PTT cells or PTT p2T7^177^-TbRab23 cells. PTT pXS219-TbRab23 single clones were selected in medium containing 25 μg/ml hygromycin, 25 μg/ml geneticin, and 1.5 μg/ml puromycin. PTT cells transgenic for both TbRab23 RNAi and expression were selected and maintained as above with the addition of 3 μg/ml phleomycin.

### 2.4. Assessment of RNA interference (RNAi) on proliferation

RNAi was induced in log-phase parasites by the addition of tetracycline at 1 μg/ml. Silencing of the target was monitored by Western blotting on cell lines expressing epitope-tagged TbRab23. BSF proliferation curves were performed in triplicate by inoculating cultures at 1 × 10^4 ^cells/ml each day in fresh medium with antibiotics. Cell densities were determined using a Coulter Z1 Counter (Beckman). PCF proliferation curves were performed in triplicate by inoculating cultures at 5 × 10^5 ^cells/ml every two days in fresh medium with antibiotics.

### 2.5. Western blot analysis

Cells were harvested at 800 g for 10 minutes and washed twice in ice-cold phosphate buffered saline (PBS) (Sigma). 10^7 ^cells were heated in 2× SDS-PAGE loading buffer (100 mM Tris-HCl, pH 6.8, 20% v/v glycerol, 0.2% v/v β-mercaptoethanol, 0.2% w/v bromophenol blue, 4% w/v SDS) for 5 minutes at 94°C. 10^7 ^cells per lane were loaded and resolved by 12.5% SDS-PAGE. Proteins were electrophoretically transferred onto polyvinylidene fluoride membranes (Millipore). Membranes were blocked and processed following standard procedures. Polyclonal anti-TbBiP serum (a kind gift from J. D. Bangs, University of Wisconsin) was used at 1:20000, polyclonal anti-GFP (a gift from M. Rout, Rockefeller University) 1:10000, polyclonal anti-TbRab11 1:1000, monoclonal anti-PFR (L8C4, a gift from K. Gull, University of Oxford) 1:1000, monoclonal anti-β tubulin (Chemicon) 1:10000, TbRab23 peptide sera 1:1000, and monoclonal anti-HA (Roche) 1:5000. Incubations with the appropriate commercial secondary anti-IgG horseradish peroxidase (HRP) conjugates (Sigma) were performed at 1:20000 in 1x Tris-buffered saline (with 0.1% tween 20) for 45 minutes. Detection was by chemiluminescence with luminol (Sigma) on BioMaxMR film (Kodak).

### 2.6. Subcellular fractionation

Flagella isolation was performed as previously described [32]. Briefly, 1 × 10^8 ^cells were lysed in PMN buffer (10 mM NaPO_4_, pH 7.2, 150 mM NaCl, 1 mM MgCl_2_, mini-complete, EDTA-free, protein inhibitor tablets (Roche), 0.5% Triton X-100 (v/v)) and subpellicular microtubules depolymerized by treatment with PMN buffer supplemented with 1 M sodium chloride. Flagella were isolated by centrifugation at 16000 g for 10 minutes. Supernatant fractions were concentrated by TCA precipitation using standard procedures and each fraction was subjected to SDS-PAGE and immunoblotting. Whole cell equivalents were loaded. All centrifugation and incubation steps were carried out at 4°C.

### 2.7. Quantitative real time RT-PCR

1 × 10^8 ^BSF and 5 × 10^7 ^PCF cells were harvested at 800 g for 10 minutes at 4°C and total RNA extracted using the RNeasy mini kit (Qiagen) according to the manufacturer's instructions. cDNA was generated from 2 μg RNA using a SuperscriptTM II RNaseH reverse transcriptase kit (Invitrogen). Quantitative RT-PCR was performed using iQ-SYBRGreen Supermix on a Mini Opticon Real-time PCR Detection System (BioRad) and quantified using Opticon3 software (BioRad). β-tubulin is expressed at similar levels between BSF and PCF cells [33] and used to normalize RNA input. A melting curve and gel electrophoresis was performed on amplified products to ensure amplification of the desired fragment. The following primers were used: TbRab23 F-AGTGGCTGAAGAGGTGGAGA, TbRab23 R-CACATTGCTGCCACGTACTC, β-tubulin F-CAAGATGGCTGTCACCTTCA and β-tubulin R-GCCAGTGTACCAGTGCAAGA.

### 2.8. Indirect Immunofluorescence

Cells were grown to log phase, fixed in 3% (w/v) paraformaldehyde in PBS on ice and adhered to poly-L-lysine slides. Cells were permeabilized by incubating in 0.1% (v/v) Triton-X-100, washed and blocked in 20% v/v foetal calf serum. Slides were incubated with primary antibodies for 1 hour and washed. Secondary antibodies were applied at 1:1000 for 1 hour at ambient temperature. Slides were mounted with Vectashield containing 4',6-diamidino-2-phenylindole (DAPI) to stain DNA (Vectalabs) and visualized on a Nikon Eclipse E600 epifluorescence microscope fitted with a Hamamatsu CCD digital camera. Image acquisition was performed with Metamorph software (Molecular Devices) and processing with Photoshop (Adobe). All images were taken under non-saturating conditions. All quantitation was done using identical exposures as appropriate and using the raw data within Metamorph. Subsequent image processing was performed for presentation purposes with Photoshop; representative images are shown. Antibodies were used at the following dilutions: TbPFR (L8C4) 1:50, FAZ (L3B2, a gift from K. Gull) 1:5, anti-HA 1:2000, and anti-GFP 1:2500. Isolated flagella were prepared for immunofluorescence analysis in solution, omitting the permeabilization step, and were spotted onto slides after the staining protocol was complete.

### 2.9. Immunogold electron microscopy

1x10^8 ^BSF parasites expressing TbRab23YFP were harvested by centrifugation for 10 minutes at 800 g and washed with saline (0.1 M HEPES pH 7.0, 0.98% sodium chloride). Cells were fixed in 2% formaldehyde and 0.05% glutaraldehyde in 0.1 M PIPES for 1 hour. After 3 washes in 0.1 M HEPES pH 7.0, the cell pellet was infiltrated overnight in 2.3 M sucrose (in PBS) at 4°C. Specimens were frozen in liquid nitrogen and 65 nm ultrathin cryosections cut on a Leica UCT ultramicrotome with EM FCS cryoattachment. Sections were mounted on grids with formvar support, blocked with 2 mM glycine (in PBS) followed by 10%FCS (in PBS). Anti-GFP antibody was applied for 1 hour, and the samples washed three times in PBS (5 minutes each). 15 nm Protein A gold was then applied for 30 minutes followed by three rinses in PBS. Six further washes were performed in dH_2_O over a 5-minute period and specimens were prepared for visualization by applying uranyl acetate in methylcellulose on ice for 10 minutes. Sections were viewed using a Philips CM100 electron microscope (FEI-Philips) operated at 80 Kv.

### 2.10 Ethics statement

All materials used in this study were obtained from accredited sources who operate ethical business practices.

## 3. Results

### 3.1. Evolutionary history of Rab23

**Figure 1:**
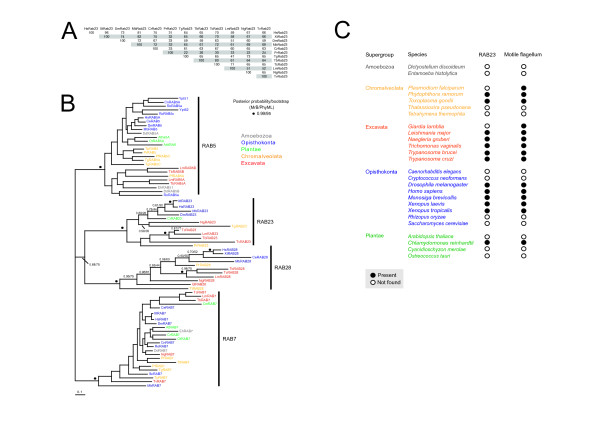
**Rab23 is monophyletic and restricted to organisms with conventional flagella**. (A) Percent identity of Rab23 homologues. T-coffee was used to produce an alignment of Rab23 sequences using default parameters. A table of percent identities was generated using the alignment http://imed.med.ucm.es/Tools/sias.html. Species abbreviations: Hs = Homo sapiens, Xt = Xenopus tropicalis, Dm = Drosophila melanogaster, Mb = Monosiga brevicolli, Cr = Chlamydomonas reinhardtii, Tb = Trypanosoma brucei, Tc = Trypanosoma cruzi, Lm = Leishmania major, Ng = Naegleria gruberi, Tv = Trichomonas vaginalis, Pr = Phytophthora ramorum and Tg = Toxoplasma gondii. (B) Phylogenetic reconstruction of Rab5, Rab7, Rab23 and Rab28 families from eukaryotes in the major sampled supergroups. Rab23 robustly forms a separate clade with Bayesian posterior probability support values greater than 0.95 denoted by a vertical bar next to the clade. Rab5 and Rab7 were included in the analysis as outgroups and subclade support values are not shown. Rab7 was used to root the tree. Circles above nodes indicate statistical support for MrBayes/PhyML 0.99/95 (posteriorprobability/bootstrap, respectively). Those nodes supported by both methods, but with lower support values are written above the node. The diversity of eukaryotic supergroups within each family is illustrated by color-coded taxon names. Species abbreviations: Hs = Homo sapiens, Xt = Xenopus tropicalis, Dm = Drosophila melanogaster, Ce = Caenorhabditis elegans, Mb = Monosiga brevicolli, Sc = Saccharomyces cerevisiae, Cn = Cryptococcus neoformans, Ro = Rhizopus oryzae, At = Arabidopsis thaliana, Cr = Chlamydomonas reinhardtii, Ot = Ostreococcus tauri, Cm = Cyanidioschyzon merolae, Tb = Trypanosoma brucei, Tc = Trypanosoma cruzi, Lm = Leishmania major, Gl = Giardia lamblia, Ng = Naegleria gruberi, Tv = Trichomonas vaginalis, Pr = Phytophthora ramorum, Pf = Plasmodium falciparum, Tt = Tetrahymena thermophila, Tg = Toxoplasma gondii, Tp = Thalassiosira pseudonana, Dd = Dictyostelium discoideum and Eh = Entamoeba histolytica. (C) Distribution of Rab23 homologues among flagellates and non-flagellates. Correlation between possession of a motile flagellum exhibiting a 9+2 microtubule arrangement and a Rab23 orthologue was found. Data suggest that Rab23 is not found in taxa lacking motile flagella or with non-conventional flagellar assembly, i.e. P. falciparum and G. lamblia.

Previous studies suggested that Rab23 is monophyletic, but this was based on limited taxon sampling [1]. Another study explored the evolutionary history of Rab23 in more detail, but restricted their sampling to opisthokonts [10]. Thus the full representation of Rab23 across eukaryotes remains unknown. We used comparative genomics to gather Rab23 orthologues, choosing genomes for inclusion based on their taxonomic position to ensure adequate representation of the major supergroups sensu Adl [21,34]. Rab23 orthologues are present in a diverse range of taxa encompassing all supergroups except the Amoebozoa, suggesting a fundamental role in eukaryotic cells (Figure 1A). Homologues of Rab23 were also absent from fungi and no paralogous expansion was observed for any of the genomes analyzed. The absence of Rab23 is most likely indicative of secondary loss. Protozoan and opisthokont Rab23 orthologues differ greatly within the C-terminal hypervariable domain (Additional file 1), a region implicated in targeting of Rab proteins to specific organelles and conferring some functional specificity [35,36]. This may suggest differences in the location and function of Rab23 between divergent species, in contrast to the majority of Rab proteins [6].

As mammalian Rab23 has some function at the endosome [37], the relationship between Rab23 and other major endocytic Rab proteins, Rab5, Rab7 and Rab28 (Lumb et al., 2011, submitted) was investigated. Phylogenetic reconstruction of Rab23 unequivocally demonstrated that Rab23 is monophyletic with high statistical support and consistent with previous reconstructions (Figure 1B) [1,10,38]. Furthermore, correlation between the presence of a Rab23 gene and possession of a motile flagellum was found (Figure 1C). Out of 26 species sampled, 15 have motile flagella, and, bar Giardia lamblia and Plasmodium falciparum both of which have non-canonical flagella, all possess a Rab23 homologue. Rab23 orthologues were not found in any of the non-flagelated organisms, suggesting that Rab23 has flagellum-associated functions, supported by the finding that dominant negative forms of Rab23 prevent primary cilia formation in mammalian cells [19].

### 3.2. TbRab23 mRNA is constitutively expressed

**Figure 2:**
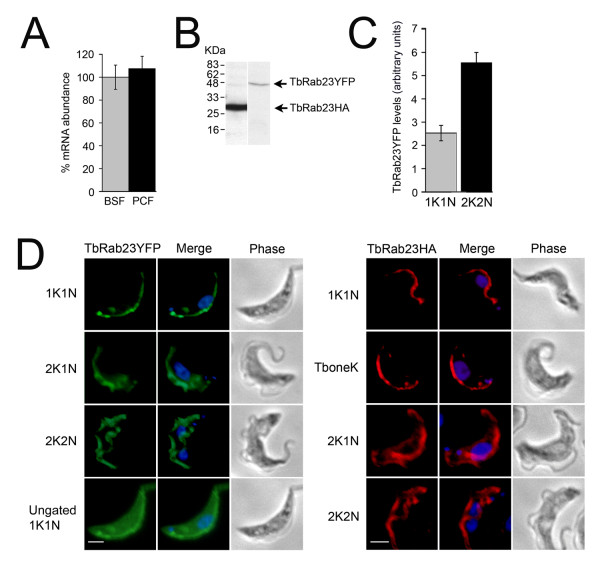
**TbRab23 localizes to the trypanosome flagellum and cell body in BSF cells**. (A) TbRab23 mRNA is expressed to equivalent levels in BSF and PCF. Three separate RNA extractions were performed on WT BSF and PCF cells and were subjected to qRT-PCR. The data were normalized for RNA input to β-tubulin and expression of TbRab23 mRNA in PCF calibrated against expression in BSF (100%). The errors bars are the standard deviation over the three biological replicates. (B) Production of an in-frame tagged polypeptide of the correct molecular weight was verified by Western blot on BSF expressing either TbRab23YFP or TbRab23HA. (C) Histogram detailing expression levels of TbRab23YFP in 1K1K and 2K2N cells by immunofluorescence on unprocessed images. TbRab23YFP levels were quantified using Metamorph software and plotted graphically. The error bars denote the s.e.m (n = 20). Grey bar represents G1 (1K1N) cells and black bar mitotic (2K2N) cells. (D) Gallery of IFA images showing expression of recombinant TbRab23 fused to an N-terminal YFP (left panel) or HA-tag (right panel) in BSF cells. Cells were labelled with either anti-GFP or anti-HA and counterstained with Alexa-Oregon Green or Alexa-568 and 4', 6-diamidino-2-phenlindole (DAPI, blue) for visualization of the nucleus and kinetoplast. 1K1N signifies cells in G1 phase and 2K1N, the start of nuclear S phase. Note the kinetoplast replicates before the nucleus (2K2N). TbRab23 chimeras locate to the cell body, an extended structure that runs along the length of the cell, and to a punctate structure that is always found in close association with the kinetoplast. During the course of cell division the TbRab23 punctum is seen to replicate prior to kinetoplast replication. Subsequently, in 2K2N cells, a new elongated structure positive for TbRab23 is seen to emerge, which is fully formed prior to the onset of cytokinesis. YFP stain was observed to become more diffuse during S phase. TbRab23HA locates to the same structures as TbRab23YFP. An additional category, TboneK, denotes the point at which a kinetoplast has replicated but has not undergone separation. All images were captured at the same exposure. For clarity, some of the images were gated to remove the cell body signal. An ungated 1K1N cell is shown for comparison. Scale bar 2 μm.

To investigate expression of the TbRab23 gene, triplicate RNA extractions were performed on BSF and PCF cells and these were subjected to quantitative real time PCR. TbRab23 transcripts are present at levels approximately 300-fold lower than β-tubulin in BSF cells, indicating that TbRab23 protein expression levels are also likely to be very low. No discernible difference in TbRab23 expression was noted between the two life stages (Figure 2A) in agreement with protein expression and microarray data [1,31].

### 3.3. Location of TbRab23

Given the increasing evidence for flagellar-associated functions for mammalian Rab23 and the evolutionary correlation between Rab23 and motile flagella, we sought to re-examine the location of TbRab23. Re-investigation of both the original antisera and a new anti-peptide sera suggested significant issues with specificity (data not shown and Additional file 2). For example, we observed high molecular weight cross-reacting bands in blots using the original antisera, which may represent detection of highly repetitive polypeptides at the nuclear envelope [39], and the peptide sera did not recognise endogenous TbRab23 by Western blot or IF. Given the low level of expression of TbRab23 as a confounding factor and questionable specificity of antisera, we chose an independent strategy to re-examine TbRab23 location.

Production of ectopic TbRab23HA (27 kDa) and TbRab23YFP (51 kDa) proteins of the correct molecular weight were verified by Western blotting (Figure 2B). The location of epitope-tagged TbRab23 was followed throughout the trypanosome cell cycle. Using an anti-GFP antibody, TbRab23YFP was localized to the cell body, an elongated structure and a punctate feature adjacent to the kinetoplast (Figure 2D, left panel). The positioning and replication of these two structures matched that of the trypanosome flagellum and basal bodies respectively. As the cell cycle proceeded, increased staining of the cell body was observed. Quantitation of fluorescence levels revealed that mitotic cells possess double the amount of TbRab23YFP protein compared to post-mitotic cells, consistent with flagellar replication (Figure 2C). The location of TbRab23HA mirrored TbRab23YFP (Figure 2D, right panel). We consider the possibility that two tags caused TbRab23 to mislocalize to the same structures to be highly unlikely. Furthermore, both of the vectors used generated modest overexpression, of the order of five-fold [40]. Similar staining has been reported for the trypanosome orthologue of IFT172 [41], which also has a role in Shh signaling in mammals [42]. Immunofluorescence (IF) using anti-HA and anti-GFP on untransfected cells did not produce fluorescence signal (data not shown). In addition, tetracycline-inducible RNA interference (RNAi) was used to validate the specificity of the HA-tag in BSF and PCF cells. Most significantly, no nuclear staining was observed with either tagged form of TbRab23 or at any point in the cell cycle. Given the small size of the HA epitope we consider it highly unlikely that this obscured a nuclear localization signal. Furthermore, fusion of GFP adjacent to the NLS of an authentic nuclear envelope protein, NUP-1, did not prevent correct nuclear targeting of that protein, suggesting that the presence of a tag does not necessarily disrupt nuclear import (data not shown).

### 3.4. TbRab23 is physically linked to basal bodies and their accessory proteins

To confirm the relationship of TbRab23 with the flagellum, cells were detergent extracted to remove cell membrane and soluble proteins, followed by depolymerization of the subpellicular array, leaving the flagellum cytoskeleton intact [32]. This procedure purifies the axoneme, flagellum attachment zone (FAZ), paraflagellar rod (PFR), four specialized microtubules and the basal body from remaining cellular components.

**Figure 3:**
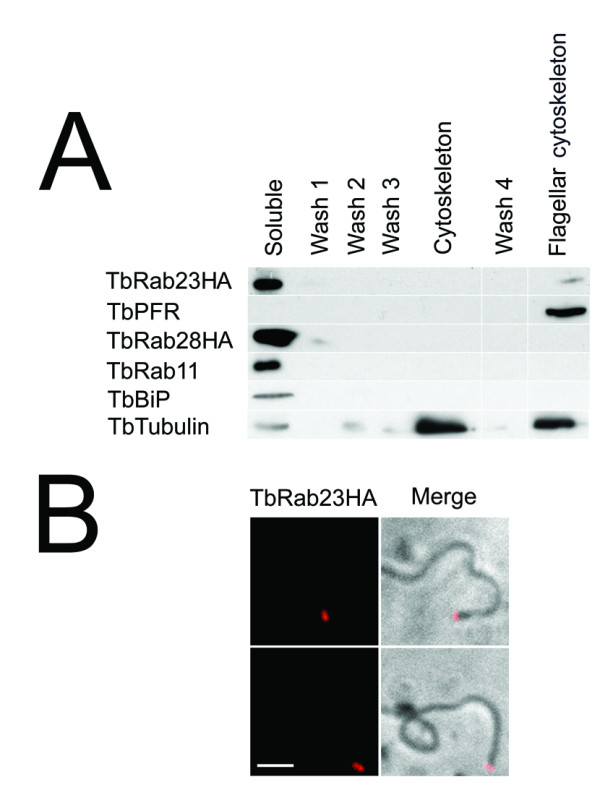
**TbRab23 is a flagellar protein**. (A) Subcellular fractionation of trypanosomes corroborates the location of tagged TbRab23. 1x10^7 ^BSF cells were incubated in PMN buffer to separate flagellar, basal body and cytoskeletal-associated proteins from the rest of the proteome ('Soluble'). 10^7 ^cell equivalents were collected at each stage for SDS-PAGE; 'Flagellar cytoskeleton' denotes pellet containing isolated axoneme, flagellum attachment zone (FAZ), paraflagellar rod (PFR), four specialized microtubules and basal body proteins. The majority of TbRab23HA was recovered in the first fraction, but a small amount was also present in the same fraction as TbPFR indicating the presence of TbRab23HA on the flagellar cytoskeleton. The cytoskeleton and flagellar cytoskeleton were enriched in β-tubulin as expected. For presentation purposes, empty wells have been removed from the figure and are represented by gaps. The isolation has been performed twice with similar results. (B) Purified flagella were processed for IF using anti-HA counterstained with Alexa-568 revealing the presence of TbRab23 at a structure associated with the flagellum on basal bodies. Scale bar 2 μm. IF on flagella extracted from cells expressing a late endosomal protein TbVps26HA, did not produce a signal (data not shown).

The majority of TbRab23HA was found in the fraction containing soluble proteins, which is common for Rab proteins. This fraction is the sum of cytosolic TbRab23 but also active TbRab23 associated with detergent-solubilized membrane via the prenylated anchor. However, additional protein was also returned in the same fraction as the paraflagellar rod, indicating that a subpopulation of TbRab23HA may also associate with the flagellar cytoskeleton (Figure 3A). Importantly TbRab11, TbRab28 and the ER chaperone TbBiP, were not associated with the flagellar cytoskeleton. As expected, tubulin was predominantly enriched in the cytoskeletal and flagellar fractions, and only 2% was found in the soluble fraction, indicating the extractions were relatively pure. Analysis of flagellar cytoskeletons by IF confirmed that only a small portion of TbRab23 remained associated with the flagellar cytoskeleton upon detergent treatment, whereas the signal along the length of the flagellum was abolished indicating that it is either membrane bound or soluble (Figure 3B).

### 3.5. Relationship of TbRab23 with TbPFR and FAZ

**Figure 4:**
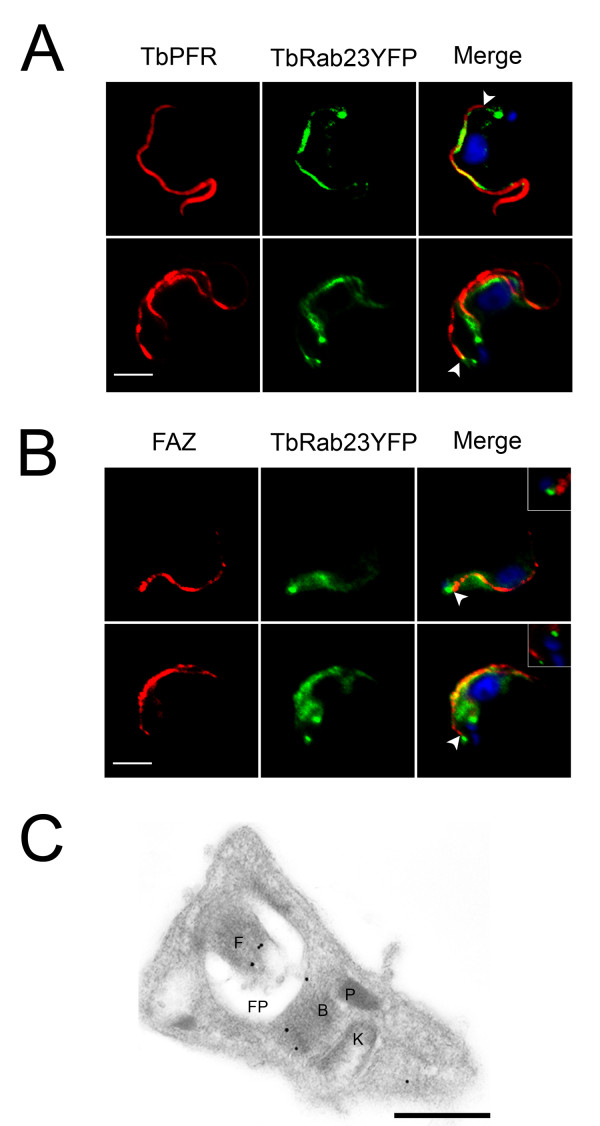
**Spatial relationship of TbRab23 to components of the flagellum**. (A) Gallery of IF images showing TbRab23YFP and PFR in BSF cells. Cells were stained with anti-GFP and anti-PFR and counterstained with Alexa-Oregon Green and Alexa-568 (red). DAPI (blue) was used to visualize DNA. Flagellum-associated TbRab23YFP is juxtaposed to PFR. Scale bar 2 μm. Arrows demark where the PFR emerges from the cell; TbRab23 staining extends beyond this point towards the flagellar pocket. For clarity images have been processed to remove cytosolic/plasma membrane staining of TbRab23. Representative images of over 20 cells are shown. (B) TbRab23YFP is coincident with flagella attachment zone protein (FAZ). The region where the flagellum attaches to the cell was visualized using antibodies raised against FAZ counterstained with Alexa-568 (red). TbRab23YFP was indirectly imaged with anti-GFP and Alexa-Oregon Green. DAPI (blue) was used to visualize DNA. Arrows demark where the FAZ originates. The insets have been gated to emphasise a pool of TbRab23YFP that is not associated with the flagellum in relation to FAZ. The proximal end of FAZ extends to the point of TbRab23 foci and was often found juxtaposed, but did not extend beyond it, indicating that TbRab23 foci at the vicinity of the basal bodies. Representative images of over 20 cells are shown. Scale bar 2 μm. (C) BSF parasites expressing TbRab23YFP labelled with anti-GFP antibodies and counterstained with gold-conjugated secondary antibody. Gold particles (15nm) were seen on the flagellum in the majority of sections analyzed (95%; n = 30), in addition to the basal body. Particles were occasionally seen between the kinetoplast and nucleus possibility defining an endosomal pool. 'B' basal body, 'F' flagellum, 'K' kinetoplast, 'P' pro-basal body, 'FP' flagellar pocket. Scale bar 200nm. Representative images are shown.

To firmly corroborate the presence of TbRab23 on the trypanosome flagellum, dual labeling with antibodies against TbPFR and the flagellum attachment zone (FAZ) were performed. TbRab23YFP was found juxtaposed to TbPFR on the length of the flagellum. In addition TbRab23YFP puncta were seen to extend approximately 0.2 μm beyond the paraflagellar rod into the cell (Figure 4A). The proximal end of the FAZ extended down to this point, but did not extend beyond the TbRab23 foci, thus suggesting that TbRab23 is present on basal bodies or structures tightly coupled to basal bodies (Figure 4B). Finally, immunoelectron microscopy was performed on transgenic BSF cells stably expressing TbRab23YFP. Cryosections revealed the presence of gold particles on basal bodies, flagella and in some cases an area posterior to the pro-basal body (Figure 4C). Overall these observations demonstrate that TbRab23 has a presence at the flagellum, as well as a soluble fraction within the cell body.

**Figure 5:**
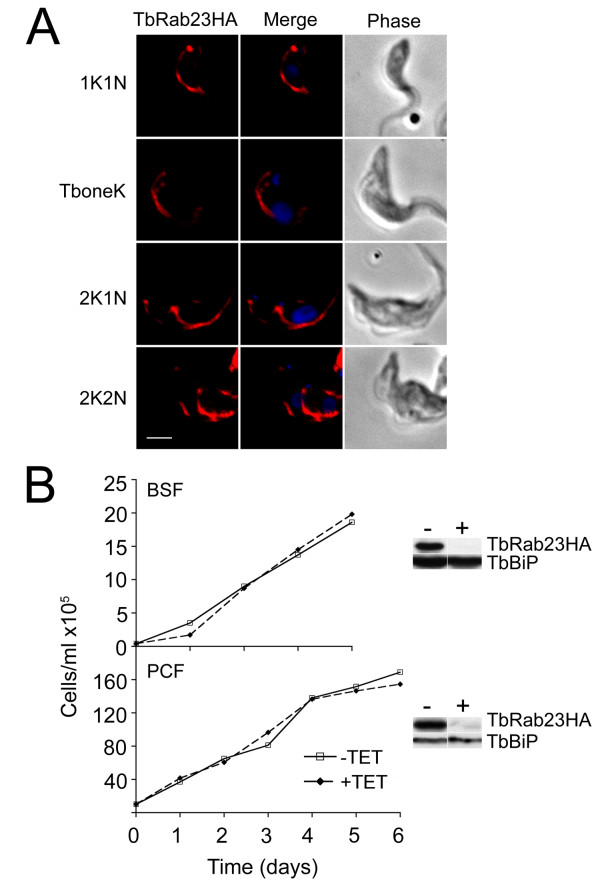
**TbRab23 location is conserved in the PCF and expression is nonessential for proliferation of BSF and PCF cells**. (A) A PCF strain constitutively expressing HATbRab23 fused to HA was stained with anti-HA and counterstained with Alexa-568 (red) to indirectly visualise TbRab23HA, together with DAPI to image the nucleus and kinetoplast. The location of TbRab23 in PCF was followed through the stages of the cell cycle, shown to the left of the images. Scale bar 2 μm. (B) Cumulative proliferation curves of BSF TbRab23^RNAi ^cells (upper panel) and PCF TbRab23^RNAi ^cells (lower panel) grown in the presence (broken line) or absence (solid line) of 1 μg/ml tetracycline. Silencing was validated at 24 hours post-induction by Western blot on RNAi cells expressing recombinant TbRab23, shown to the right of each graph. TbBiP was used to ensure accurate loading and specificity. "-" denotes uninduced and "+" induced cells.

### 3.6. TbRab23 is not required for parasite proliferation

When the equivalent TbRab23 chimeras were expressed in procyclic cells the localization was similar to the bloodstream stages, suggesting that the location of TbRab23 is conserved between developmental life forms (Figure 5A). Subsequently, the requirement for TbRab23 in parasite proliferation was examined in BSF and PCF cells by RNAi. TbRab23 was found to be non-essential in BSF cells (Figure 5B, top panel). Knockdown efficiency was validated by Western blot on a TbRab23^RNAi ^mutant ectopically expressing TbRab23HA. Expression of TbRab23 protein was rapidly suppressed by ~95% at 24 hours whilst TbBiP levels remained unchanged indicating that the RNAi construct was specific and very efficient. Examination of cells throughout induction by IF showed the transient appearance of a small population (6%) with an abnormal number of kinetoplasts and nuclei, but these cells were rapidly lost from the population (data not shown), and no significant change to cell cycle progression was recorded thereafter. No proliferation defect was noted in PCF cells (Figure 5B, bottom panel). Knockdown efficiency was validated by Western blot on a TbRab23^RNAi ^mutant ectopically expressing TbRab23HA revealing that recombinant protein levels were reduced by ~90% after 24 hours of induction.

## 4. Discussion

Rab23 was originally descibed in metazoan cells as being a component of a signaling pathway important to development, and this model is now well supported [7-11]. However, the wide taxonomic distribution of Rab23 amongst diverse unicellular organisms indicates a more fundamental cellular role for this protein beyond regulation of multicellular development, and is supported, for example, by evidence that Rab23 is not part of Shh signaling in Drosophila melanogaster [8]. Identifiable homologues of Patched or Smo are absent from trypanosomes and a great many other taxa, making the existence of a primitive Shh-like signaling network unlikely.

Comparative genomics reveals a strong correlation between possession of a motile flagellum and a Rab23 gene, supporting an early evolving flagellar-related function and also suggesting evolutionary pressure to retain Rab23 when the motile flagellum is present. Hence Rab23 has likely been repeatedly lost throughout evolution in organisms where the motile flagellum has been discarded, further supporting a model where the primary function(s) of Rab23 are flagellar-associated. G. lamblia and P. falciparum, are apparent exceptions, but both organisms possess divergent flagella; P. falciparum has lost IFT proteins and flagellum biogenesis is fully IFT-independent, whilst in G. lamblia, flagellum biogenesis is fully or partially IFT-independent [44,45].

Epitope-tagged TbRab23 localizes to the flagellum and cell body in BSF and PCF cells, and subcellular fractionation and immuno-gold labeling confirmed the presence of flagellar-associated TbRab23. These data support a role for Rab23 in flagellum function in trypanosomes, consistent with the localization of mammalian Rab23 in MDCK cells and its role in ciliary biogenesis and protein turnover [10,19,20,37]. Clearly these data are at variance with Dhir and Field (2004). However, the affinity-purified antibodies generated for this study do detect several high molecular weight antigens in trypanosome cell lysates in addition to TbRab23, indicating the presence of cross-reactive antibody. This is cause for concern as many nuclear components are high molecular weight coiled-coil proteins, including NUP-1 [39]. Moreover as NUP-1 is a high abundance repetitive protein, immunostaining may have emphasized the cross-reactive component of this and other nuclear envelope antigens.

Concurrent with a potential role in flagellar function TbRab23 exhibits features that are similar to IFT proteins. Firstly, TbRab23 extends beyond the PFR into the cell and is likely present on the flagellar membrane, as TbRab23 along the length of the flagellum is extractable by detergent, similar to many trypanosome IFT proteins [41]. Secondly, the presence of TbRab23 at the basal body is significant, as basal body transitional fibers act as docking sites for IFT particles [46,47] and many IFT proteins additionally localize to this area [48]. Thirdly, detergent extraction abolished most of the TbRab23 flagellar signal, whilst the basal body signal was retained. Differential extractability has previously been reported for IFT172 [41], IFT20 [49] and proteins of the BBSome [50]. Therefore, in addition to localization, TbRab23 shares biochemical properties with proteins important in ciliary maintenance and biogenesis. Finally, subcellular fractionation of Chlamydomonas reinhardtii flagella identified Rab23 in the membrane and matrix fraction, together with IFT A and B complexes [51]. TbRab23 was not found in a trypanosome flagellum proteome [52], presumably because flagellar membranes were stripped before analysis. However we are unable to unequivocally assign TbRab23 to the flagellum membrane or other flagellar subcompartment from the data presented here.

Surprisingly, depletion of TbRab23 did not result in a flagellar defect. This is unlikely due to inefficient suppression of protein levels, as the BSF and PCF RNAi construct effectively eliminated recombinant TbRab23 expression at levels five to ten-fold higher than endogenous protein [10]. Rather Rab23 may not be required for construction or maintenance of the flagellum in trypanosomes. Alternatively TbRab23 may exhibit functional redundancy with other IFT proteins or participate in a role that is not essential for the continuation of the cell cycle.

The data reported here indicate that trypanosome Rab23 is associated with the flagellum, and not with the inner nuclear envelope as previously suggested. Use of two distinct tags, correlation between possession of a Rab23 and a flagellum, cross-reactivity in the original sera, plus additional evidence from mammalian cell studies argues that this is the correct assignment. Hence these data unify the location of Rab23 across the eukaryotes.

## Abbreviations

BBS: Bardet-Biedl Syndrome; BiP: Binding protein; BSF: bloodstream form; FAZ: flagellum attachment zone; IFT: Intraflagellar transport; PCF: procyclic form; PFR: paraflagellar rod; Rab: Ras-related proteins in brain, Shh: Sonic hedgehog.

## Competing interests

The authors declare that they have no competing interests.

## Authors' contributions

MCF and JL designed the study, JL performed the experimental, MCF and JL analyzed the data and wrote the manuscript.

## Supplementary Material

Additional file 1 **Alignment of Rab23 orthologues**. T-coffee and ESPript were used to generate and format the alignment. Identities are white on a black background and similarities are boxed on a white background. Numbers above the residues correspond to residues within the human orthologue. Species abbreviations as before. Click here for file

Additional file 2 **Western blot analysis of anti-Rab23 antisera specificity**. Whole cell lysates of induced and uninduced Eschericia coli harboring a GST-Rab23 fusion protein expression construct, or wild type T. brucei bloodstream (BSF) cells and transgenic ectopic expressors for TbRab23HA probed with peptide antisera. Molecular weight standards are at left in kDa, and migration positions of the GST-Rab23 fusion protein and TbRab23HA are indicated at right. Note the presence of high molecular weight cross-reactive material. Click here for file

## References

[B1] Dhir V Field MC TbRAB23; a nuclear-associated Rab protein from Trypanosoma brucei Mol Biochem Parasitol 2004 136 297 301 10.1016/j.molbiopara.2003.12.014 15478808

[B2] Stenmark H Rab GTPases as coordinators of vesicle traffic Nat Rev Mol Cell Biol 2009 10 513 25 19603039 10.1038/nrm2728

[B3] Ackers JP Dhir V Field MC A bioinformatic analysis of the RAB genes of Trypanosoma brucei Mol Biochem Parasitol 2005 141 89 97 10.1016/j.molbiopara.2005.01.017 15811530

[B4] Field MC Carrington M The trypanosome flagellar pocket Nat Rev Microbiol 2009 7 775 86 10.1038/nrmicro2221 19806154

[B5] Natesan SK Peacock L Leung KF Matthews KR Gibson W Field MC The trypanosome Rab-related proteins RabX1 and RabX2 play no role in intracellular trafficking but may be involved in fly infectivity PLoS One 2009 4 7217 10.1371/journal.pone.0007217 PMC2748683 19787065

[B6] Brighouse A Dacks JB Field MC Rab protein evolution and the history of the eukaryotic endomembrane system Cell Mol Life Sci 2010 67 3449 56 10.1007/s00018-010-0436-1 20582450 PMC2943070

[B7] Eggenschwiler JT Espinoza E Anderson KV Rab23 is an essential negative regulator of the mouse Sonic hedgehog signaling pathway Nature 2001 412 194 8 10.1038/35084089 11449277

[B8] Hooper JE Scott MP Communicating with Hedgehogs Nat Rev Mol Cell Biol 2005 6 306 17 10.1038/nrm1622 15803137

[B9] Evans TM Simpson F Parton RG Wicking C Characterization of Rab23, a negative regulator of sonic hedgehog signaling Methods Enzymol 2005 403 759 77 16473637 10.1016/S0076-6879(05)03066-1

[B10] Guo A Wang T Ng EL Aulia S Chong KH Teng FY Wang Y Tang BL Open brain gene product Rab23: expression pattern in the adult mouse brain and functional characterization J Neurosci Res 2006 83 1118 27 10.1002/jnr.20788 16463280

[B11] Eggenschwiler JT Bulgakov OV Qin J Li T Anderson KV Mouse Rab23 regulates hedgehog signaling from smoothened to Gli proteins Dev Biol 2006 290 1 12 10.1016/j.ydbio.2005.09.022 16364285

[B12] McMahon AP Ingham PW Tabin CJ Developmental roles and clinical significance of hedgehog signaling Curr Top Dev Biol 2003 53 1 114 12509125 10.1016/s0070-2153(03)53002-2

[B13] Varjosalo M Taipale J Hedgehog: functions and mechanisms Genes Dev 2008 22 2454 72 10.1101/gad.1693608 18794343

[B14] Haycraft CJ Banizs B Aydin-Son Y Zhang Q Michaud EJ Yoder BK Gli2 and Gli3 localize to cilia and require the intraflagellar transport protein polaris for processing and function PLoS Genet 2005 1 e53 10.1371/journal.pgen.0010053 16254602 PMC1270009

[B15] Corbit KC Aanstad P Singla V Norman AR Stainier DY Reiter JF Vertebrate Smoothened functions at the primary cilium Nature 2005 437 1018 21 10.1038/nature04117 16136078

[B16] Rohatgi R Milenkovic L Scott MP Patched1 regulates hedgehog signaling at the primary cilium Science 2007 317 372 6 10.1126/science.1139740 17641202

[B17] Kovacs JJ Whalen EJ Liu R Xiao K Kim J Chen M Wang J Chen W Lefkowitz RJ Beta-arrestin-mediated localization of smoothened to the primary cilium Science 2008 320 1777 81 10.1126/science.1157983 18497258 PMC2587210

[B18] Tran PV Haycraft CJ Besschetnova TY Turbe-Doan A Stottmann RW Herron BJ Chesebro AL Qiu H Scherz PJ Shah JV Yoder BK Beier DR THM1 negatively modulates mouse sonic hedgehog signal transduction and affects retrograde intraflagellar transport in cilia Nat Genet 2008 40 403 10 10.1038/ng.105 18327258 PMC4817720

[B19] Yoshimura S Egerer J Fuchs E Haas AK Barr FA Functional dissection of Rab GTPases involved in primary cilium formation J Cell Biol 2007 178 363 9 10.1083/jcb.200703047 17646400 PMC2064854

[B20] Boehlke C Bashkurov M Buescher A Krick T John AK Nitschke R Walz G Kuehn EW Differential role of Rab proteins in ciliary trafficking: Rab23 regulates smoothened levels J Cell Sci 2010 123 1460 7 10.1242/jcs.058883 20375059

[B21] Roger AJ The real 'kingdoms' of eukaryotes Curr Biol 2004 14 R693 6 10.1016/j.cub.2004.08.038 15341755

[B22] Altschul SF Madden TL Schaffer AA Zhang J Zhang Z Miller W Lipman DJ Gapped BLAST and PSI-BLAST: a new generation of protein database search programs Nucleic Acids Res 1997 25 3389 402 10.1093/nar/25.17.3389 9254694 PMC146917

[B23] Larkin MA Blackshields G Brown NP Chenna R McGettigan PA McWilliam H Valentin F Wallace IM Wilm A Lopez R Thompson JD Gibson TJ Higgins DG Clustal W and Clustal × version 2.0 Bioinformatics 2007 23 2947 8 10.1093/bioinformatics/btm404 17846036

[B24] Maddison WP Maddison DR Interactive analysis of phylogeny and character evolution using the computer program MacClade Folia Primatol 1989 53 190 202 10.1159/000156416 2606395

[B25] Abascal F Zardoya R Posada D ProtTest: Selection of best-fit models of protein evolution Bioinformatics 2005 21 9 2104 2105 10.1093/bioinformatics/bti263 15647292

[B26] Whelan S Goldman N A general empirical model of protein evolution derived from multiple protein families using a maximum-likelihood approach Mol Biol Evol 2001 18 691 699 11319253 10.1093/oxfordjournals.molbev.a003851

[B27] Field H Field MC Tandem duplication of rab genes followed by sequence divergence and acquisition of distinct functions in Trypanosoma brucei J Biol Chem 1997 272 10498 505 10.1074/jbc.272.16.10498 9099693

[B28] Wirtz E Leal S Ochatt C Cross GA A tightly regulated inducible expression system for conditional gene knock-outs and dominant-negative genetics in Trypanosoma brucei Mol Biochem Parasitol 1999 99 89 101 10.1016/S0166-6851(99)00002-X 10215027

[B29] Bastin P MacRae TH Francis SB Matthews KR Gull K Flagellar morphogenesis: protein targeting and assembly in the paraflagellar rod of trypanosomes Mol Cell Biol 1999 19 8191 200 10567544 10.1128/mcb.19.12.8191 PMC84903

[B30] Redmond S Vadivelu J Field MC RNAit: an automated web-based tool for the selection of RNAi targets in Trypanosoma brucei Mol Biochem Parasitol 2003 128 115 8 10.1016/S0166-6851(03)00045-8 12706807

[B31] Koumandou VL Natesan SK Sergeenko T Field MC The trypanosome transcriptome is remodelled during differentiation but displays limited responsiveness within life stages BMC Genomics 2008 9 298 10.1186/1471-2164-9-298 18573209 PMC2443814

[B32] Sherwin T Gull K The cell division cycle of Trypanosoma brucei brucei: timing of event markers and cytoskeletal modulations Philos Trans R Soc Lond B Biol Sci 1989 323 573 88 10.1098/rstb.1989.0037 2568647

[B33] Diehl S Diehl F El-Sayed NM Clayton C Hoheisel JD Analysis of stage-specific gene expression in the bloodstream and the procyclic form of Trypanosoma brucei using a genomic DNA-microarray Mol Biochem Parasitol 2002 123 115 23 10.1016/S0166-6851(02)00138-X 12270627

[B34] Adl SM Simpson AG Farmer MA Andersen RA Anderson OR Barta JR Bowser SS Brugerolle G Fensome RA Fredericq S James TY Karpov S Kugrens P Krug J Lane CE Lewis LA Lodge J Lynn DH Mann DG McCourt RM Mendoza L Moestrup O Mozley-Standridge SE Nerad TA Shearer CA Smirnov AV Spiegel FW Taylor MF The new higher level classification of eukaryotes with emphasis on the taxonomy of protists J Eukaryot Microbiol 2005 52 399 451 10.1111/j.1550-7408.2005.00053.x 16248873

[B35] Chavrier P Gorvel JP Stelzer E Simons K Gruenberg J Zerial M Hypervariable C-terminal domain of rab proteins acts as a targeting signal Nature 1991 353 769 72 10.1038/353769a0 1944536

[B36] Ali BR Seabra MC Targeting of Rab GTPases to cellular membranes Biochem Soc Trans 2005 33 652 6 10.1042/BST0330652 16042566

[B37] Evans TM Ferguson C Wainwright BJ Parton RG Wicking C Rab23, a negative regulator of hedgehog signaling, localizes to the plasma membrane and the endocytic pathway Traffic 2003 4 869 84 10.1046/j.1600-0854.2003.00141.x 14617350

[B38] Pereira-Leal JB Seabra MC Evolution of the Rab family of small GTP-binding proteins J Mol Biol 2001 313 889 901 10.1006/jmbi.2001.5072 11697911

[B39] Rout MP Field MC Isolation and characterization of subnuclear compartments from Trypanosoma brucei. Identification of a major repetitive nuclear lamina component J Cell Bio 2001 276 38261 71 10.1074/jbc.M104024200 11477078

[B40] Pal A Hall BS Jeffries TR Field MC Rab5 and Rab11 mediate transferrin and anti-variant surface glycoprotein antibody recycling in Trypanosoma brucei Biochem J 2003 374 443 51 10.1042/BJ20030469 12744719 PMC1223594

[B41] Absalon S Blisnick T Kohl L Toutirais G Dore G Julkowska D Tavenet A Bastin P Intraflagellar transport and functional analysis of genes required for flagellum formation in trypanosomes Mol Biol Cell 2008 19 929 44 18094047 10.1091/mbc.E07-08-0749 PMC2262991

[B42] Huangfu D Liu A Rakeman AS Murcia NS Niswander L Anderson KV Hedgehog signaling in the mouse requires intraflagellar transport proteins Nature 2003 426 83 7 10.1038/nature02061 14603322

[B43] Briggs LJ Davidge JA Wickstead B Ginger ML Gull K More than one way to build a flagellum: comparative genomics of parasitic protozoa Curr Biol 2004 14 R611 2 10.1016/j.cub.2004.07.041 15296774

[B44] Jekely G Arendt D Evolution of intraflagellar transport from coated vesicles and autogenous origin of the eukaryotic cilium Bioessays 2006 28 191 8 10.1002/bies.20369 16435301

[B45] Benchimol M Trichomonads under Microscopy Microsc Microanal 2004 10 528 50 15525428 10.1017/S1431927604040905

[B46] Rosenbaum JL Witman GB Intraflagellar transport Nat Rev Mol Cell Biol 2002 3 813 25 10.1038/nrm952 12415299

[B47] Deane JA Cole DG Seeley ES Diener DR Rosenbaum JL Localization of intraflagellar transport protein IFT52 identifies basal body transitional fibers as the docking site for IFT particles Curr Biol 2001 11 1586 90 10.1016/S0960-9822(01)00484-5 11676918

[B48] Cole DG Diener DR Himelblau AL Beech PL Fuster JC Rosenbaum JL Chlamydomonas kinesin-II-dependent intraflagellar transport (IFT): IFT particles contain proteins required for ciliary assembly in Caenorhabditis elegans sensory neurons J Cell Biol 1998 141 993 1008 10.1083/jcb.141.4.993 9585417 PMC2132775

[B49] Follit JA Tuft RA Fogarty KE Pazour GJ The intraflagellar transport protein IFT20 is associated with the Golgi complex and is required for cilia assembly Mol Biol Cell 2006 17 3781 92 10.1091/mbc.E06-02-0133 16775004 PMC1593158

[B50] Nachury MV Loktev AV Zhang Q Westlake CJ Peranen J Merdes A Slusarski DC Scheller RH Bazan JF Sheffield VC Jackson PK A core complex of BBS proteins cooperates with the GTPase Rab8 to promote ciliary membrane biogenesis Cell 2007 129 1201 13 10.1016/j.cell.2007.03.053 17574030

[B51] Pazour GJ Agrin N Leszyk J Witman GB Proteomic analysis of a eukaryotic cilium J Cell Biol 2005 170 103 13 10.1083/jcb.200504008 15998802 PMC2171396

[B52] Broadhead R Dawe HR Farr H Griffiths S Hart SR Portman N Shaw MK Ginger ML Gaskell SJ McKean PG Gull K Flagellar motility is required for the viability of the bloodstream trypanosome Nature 2006 440 224 7 10.1038/nature04541 16525475

