# Supplementary figures and images for: Rab23 is a flagellar protein in Trypanosoma brucei
Source: BMC Res Notes. 2011 Jun 15;4:190. doi: 10.1186/1756-0500-4-190 (PMC3138460; doi:10.1186/1756-0500-4-190)

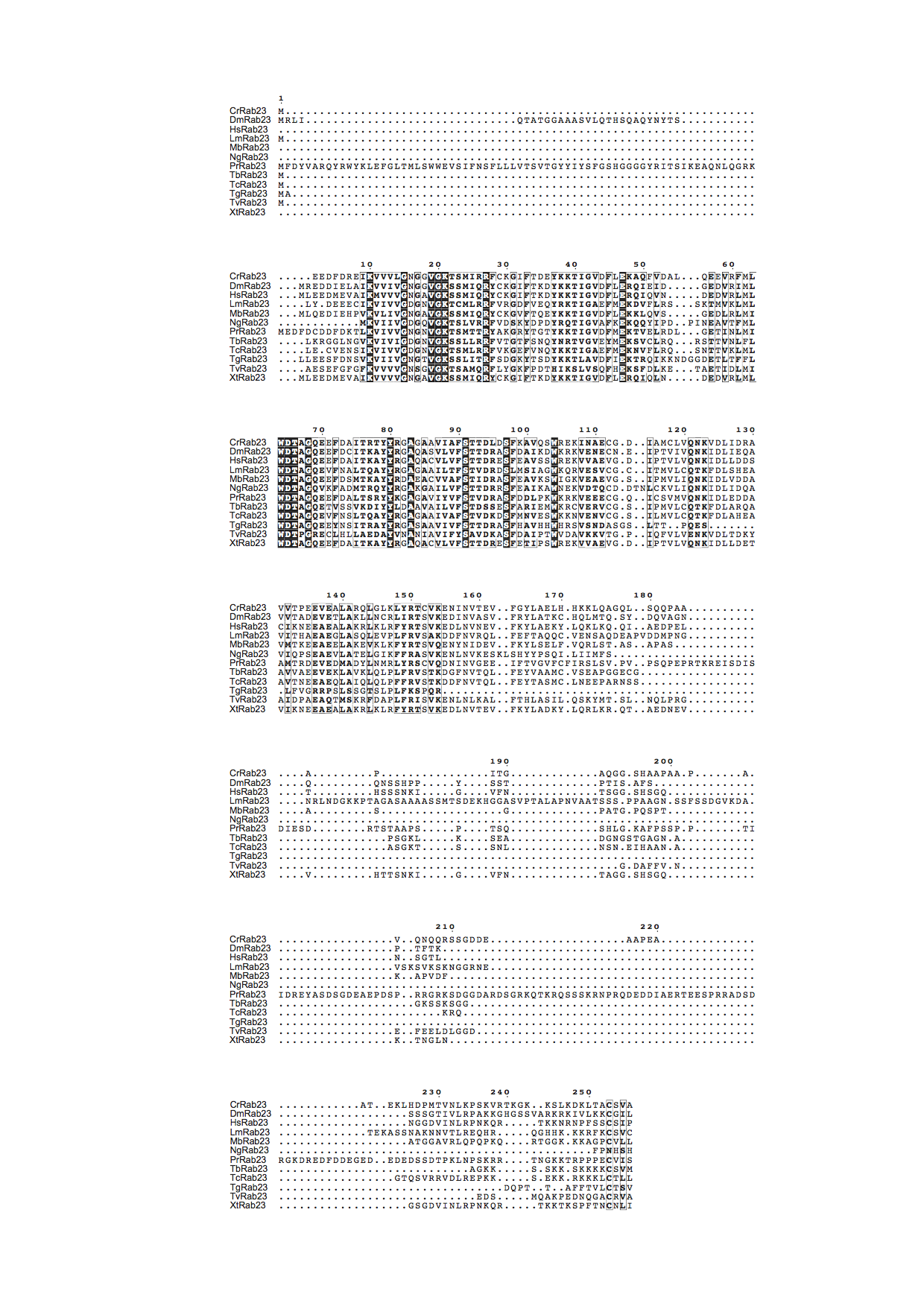

Supplement: Additional file 1 — Alignment of Rab23 orthologues. T-coffee and ESPript were used to generate and format the alignment. Identities are white on a black background and similarities are boxed on a white background. Numbers above the residues correspond to residues within the human orthologue. Species abbreviations as before. [file 1756-0500-4-190-S1.TIFF]

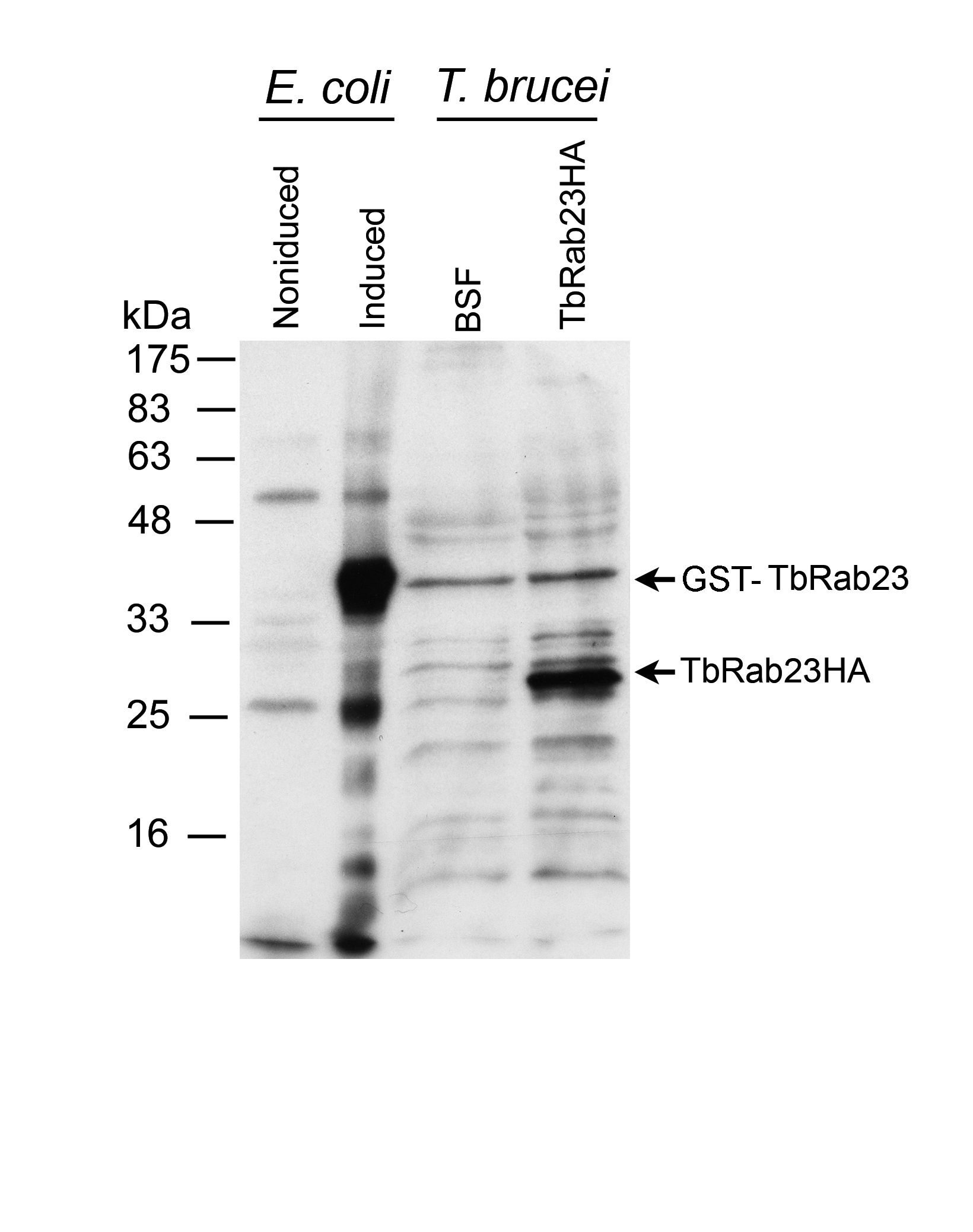

Supplement: Additional file 2 — Western blot analysis of anti-Rab23 antisera specificity. Whole cell lysates of induced and uninduced Eschericia coli harboring a GST-Rab23 fusion protein expression construct, or wild type T. brucei bloodstream (BSF) cells and transgenic ectopic expressors for TbRab23HA probed with peptide antisera. Molecular weight standards are at left in kDa, and migration positions of the GST-Rab23 fusion protein and TbRab23HA are indicated at right. Note the presence of high molecular weight cross-reactive material. [file 1756-0500-4-190-S2.TIFF]
